# Genome-wide identification and systematic characterization of *MATH* family genes and expression analysis of response to abiotic stresses in *Brassica napus L*

**DOI:** 10.1186/s12870-026-09319-7

**Published:** 2026-08-15

**Authors:** Fatima Maliha, Wenyu Wu, Linsiwan Long, Yiming Feng, Xiang Liu, Yesmagul Balziya, Tayeb Muhammad, Keqi Li, Xiaoyan Li, Xue Shi, Weinan Sun, Shimeng Li, Zhen Huang

**Affiliations:** 1 College of Agronomy, Northwest A&F University, Yangling, 712100 China; 2 Agricultural Research Institute, Xizang Academy of Agriculture and Animal Husbandry Sciences, Lhasa, 850000 China; 3 Key Laboratory of Genome Research and Genetic Improvement of Xinjiang Characteristic Fruits and Vegetables, Institute of Fruits and Vegetables, Xinjiang Academy of Agricultural Sciences, Urumqi, China

**Keywords:** *Brassica napus*, *BnMATH* family genes, Cis-acting regulatory elements, miRNA, Abiotic stresses

## Abstract

**Background:**

Meprin and TRAF-C homology (MATH) proteins function as adaptor molecules and components of E3 ubiquitin ligase complexes. They link receptor-like kinase signalling to downstream regulatory pathways in plants. MATH proteins coordinate growth, hormone signalling, and responses to abiotic stress by modulating ubiquitin-dependent proteostasis. Despite their importance, however, little is known about the diversity, function, and specific regulatory role of the *MATH* gene family in *Brassica napus*.

**Results:**

We identified 151 *BnMATH* family genes distributed across the 19 chromosomes of *B. napus* using a hidden Markov model-based genome-wide search followed by domain validation. Comparative phylogenetic and structural analyses classified these genes into four conserved clades, revealing that extensive segmental and tandem duplication events had driven the family expansion. Promoter analysis revealed more than 6,000 cis-acting regulatory elements associated with hormone- and stress-responsive gene expression. A total of 44 miRNA families targeting BnMATH genes were identified, among which 10 have been previously validated to be involved in biological processes. Transcriptome profiling combined with qRT-PCR validation revealed pronounced tissue-specific and abiotic-stress-responsive expression patterns. Notably, *BnMATH06*, *BnMATH92,* and *BnMATH135* were strongly induced by salt and drought stress, suggesting a potential role in stress adaptation.

**Conclusion:**

These findings deepen our understanding of the *MATH* gene family and provide a robust foundation for future functional genomics research targeting their specific biological roles, particularly in hormone-driven regulation and adaptation to abiotic stress.

**Supplementary Information:**

The online version contains supplementary material available at https://doi.org/10.1186/s12870-026-09319-7.

## Introduction

*B. napus L*. (genome AACC) is an allopolyploid crop that originated approximately 7,500 years ago through the natural hybridization of *B. rapa* (AA) and *B. oleracea* (CC) [1]. It is the second most important oilseed crop globally, accounting for 13.2% of world oilseed production. In 2022, 87.22 million tons were produced. The European Union is the leading producer, accounting for 29.7 million tons (35% of the global output), followed by Canada and China. Rapeseed oil has a beneficial fatty acid composition enriched with monounsaturated and polyunsaturated fatty acids and low saturated fat content. It is also abundant in tocopherols, sterols, and phytosterols, which provide nutritional and cardioprotective benefits. Beyond its use in the diet, rapeseed is a key source of feedstock for biodiesel production in Europe, accounting for 68–77% of the total supply. It is also used as a renewable raw material for bio-based products, including lubricants, resins, and polymers. The defatted meal is a valuable protein-rich livestock feed that ranks second only to soybean meal in global utilization [2].

Despite its economic and agronomic importance, rapeseed productivity is highly vulnerable to a convergence of biotic and abiotic factors. Fungal pathogens, insect pests, drought, salinity, cold, and heat collectively impose severe constraints on growth and crop yield, resulting in multi-billion-dollar economic losses each year. These constraints have intensified due to ongoing climate change, and a 30% yield reduction is projected by 2050, coinciding with a projected 50% increase in global food demand [3]. Rapeseed is particularly susceptible to environmental fluctuations, which compromise seed quality and reduce overall productivity. Therefore, enhancing stress resilience requires identifying key regulatory genes and integrating advanced molecular breeding, transgenic, and genome-editing approaches to accelerate the development of climate-resilient cultivars.

Meprin and TRAF-C homology (*MATH*) proteins are the largest subgroup within the tumor necrosis factor (*TRAF*) superfamily. They have recently been identified as key regulators of plant developmental and stress adaptation processes [4]. *MATH* genes function as adaptor molecules or RING-type E3 ubiquitin ligases, integrating receptor-like kinase signalling with downstream modules and linking external stimuli to the intracellular regulatory network. Structurally, TRAF proteins consist of a conserved C-terminal TRAF domain that contains an oligomerization domain responsible for the N-terminal coiled-coil (TRAF-N) structure, as well as a C-terminal TRAF-C subdomain. The 180-amino-acid-long TRAF-C module adopts a β-sandwich configuration composed of seven to eight antiparallel β-strands, forming a modular platform for protein–protein interactions and effector recruitment [5]. In plants, this configuration enables the assembly of dynamic multiprotein signalling complexes that regulate the stability of key regulators in important hormone pathways, including those for abscisic acid, brassinosteroids, and jasmonates. By integrating membrane-proximal perception with ubiquitin-dependent proteostasis, MATH proteins act as central nodes that coordinate growth and defence systems in response to fluctuating environmental conditions.

Meprins are membrane-anchored and secreted astacin metalloproteases that are characterized by a C-terminal region that is structurally homologous to the tumor necrosis factor receptor-associated factor (TRAF) domain. This resemblance gave rise to the term Meprin-TRAF-C homology (MATH) [6]. MATH-encoding proteins in eukaryotes often have additional domains, including BTB broad-complex, tramtrack, and bric-à-brac (BTB), poxvirus and zinc finger (POZ), ubiquitin-specific protease (UBP), really interesting new gene (RING), and filament domains [7]. The modularity of these domains enables TRAF/MATH proteins to act as scaffolds in higher-order signalling assemblies. They also regulate ubiquitin-mediated post-translational modifications, particularly ubiquitination and deubiquitination. The structural and functional conservation of the TRAF/MATH domain in facilitating proteolytic signalling and adaptor-driven complex formation emphasizes its evolutionary importance in linking receptor activation with proteostasis and response pathways [8].

The number of TRAF/MATH protein family members varies among the plant species. High-throughput sequencing enables the systematic identification of MATH domain proteins across diverse plant taxa [4, 9, 10]. Some plant genomes encode more than seventy TRAF/MATH members that function as key regulators of autophagy, gametophyte development, hormonal signalling, and environmental stress responses, positioning these proteins as molecular hubs that integrate developmental processes with stress adaptation mechanisms [10, 11].

Although significant progress has been made in characterizing MATH-domain proteins in diploid plant species such as *Arabidopsis thaliana*, *Brassica rapa*, rice, maize, and members of the *Solanaceae* family, a comprehensive, genome-wide investigation of this gene family in the allotetraploid crop *B. napus* has yet to be conducted. In this study, we systematically identified and characterized 151 *BnMATH* genes distributed across the rapeseed genome. This number represents a substantial expansion compared to that of previously studied diploid species, reflecting polyploidization-driven gene retention and diversification. We also examined their genomic organization, chromosomal distribution, evolutionary relationships, cis-regulatory architecture, and expression patterns in different tissues and under various phytohormone and abiotic stress conditions. Collectively, these findings provide the first comprehensive characterization of the *BnMATH* gene family in *B. napus*, establishing a robust genomic foundation for functional genomic studies and facilitating the identification of stress-responsive candidates to improve rapeseed's climate resilience.

## Methodology

### Identification and physiochemical profiling of the *MATH *gene family

Candidate *MATH* gene family sequences were retrieved from the *B. napus* reference genome database (http://yanglab.hzau.edu.cn/BnIR/genome-data; accessed January 12, 2025). A Hidden Markov Model (HMM) profile of the conserved MATH domain (PF00917) was downloaded from the Pfam database 38.1 (https://pfam.xfam.org; accessed January 15, 2025) and used as a query in HMMER 3.3.2 searches (https://www.ebi.ac.uk/Tools/hmmer; accessed January 18, 2025) to detect the putative candidates. The obtained protein sequences were further validated using hmmscan and the SMART genome resource (https://smart.embl.de; accessed January 20, 2025) to confirm the presence of the intact MATH domains. Redundant sequences were then removed. Key physiochemical properties, such as amino acid composition, molecular weight, theoretical isoelectric point (pI), instability index, and grand average of hydropathicity (GRAVY), were analyzed using the ProtParam tool (http://www.expasy.org/tools/protparam.html; accessed January 24, 2025). The subcellular localizations of BnMATH proteins were predicted using CELLO v2.5 (https://cello.nctu.edu.tw; accessed January 24, 2025). Full-length BnMATH protein sequences were submitted in FASTA format. During prediction, Eukaryotes was selected as the organism type and Protein was selected as the sequence type. The analysis was performed using the default parameters of CELLO v2.5, and the predicted localization results were recorded.

### Phylogenetic analysis

A comparative phylogenetic analysis was performed to investigate the evolutionary relationships of the *B. napus MATH* gene family using homologous MATH protein sequences from *Arabidopsis thaliana*. Full-length protein sequences of BnMATH and *A. thaliana* MATH proteins were aligned using the MUSCLE algorithm in MEGA 11 with the default alignment parameters. The phylogenetic tree was constructed in MEGA 11 using the Neighbor-Joining (NJ) method with the Poisson substitution model. Gaps and missing data were handled using pairwise deletion. The reliability of the tree topology was evaluated using 1,000 bootstrap replicates [12]. The resulting phylogenetic tree was visualized and refined using the Interactive Tree of Life (iTOL) web server (http://itol.embl.de/).

### Gene structure, organization, and conserved motif analysis

The *MATH* gene structural architecture of *B. napus* was analyzed by aligning coding sequences (CDS) with their corresponding genomic sequences to identify the exon–intron arrangement, using the Gene Structure Display Server (GSDS) (http://gsds.cbi.pku.edu.cn; accessed January 26, 2025). Conserved motifs in the MATH protein family were detected using the MEME Suite 5.5.9 (http://memesuite.org/tools/meme; accessed January 26, 2025), with the following parameters: motif width of 6–50 amino acids and a maximum of 20 motifs. Gene architectures and motif distributions were subsequently visualized using the Gene Structure View module in TBtools [13].

### Chromosomal mapping and synteny analysis

The chromosomal locations of *B. napus MATH* genes were identified through MG2C (http://mg2c.iask.in/mg2c_v2.1). The chromosome measurements and gene positions were retrieved from the genome annotation (GFF3) file and analyzed in TBtools. Patterns of gene duplication were visualized using Circos [14]. To investigate the duplication events underlying the evolutionary expansion of the *MATH* gene family, collinearity analyses were performed using MCScanX with the default parameters. The syntenic relationships between *A. thaliana* and *B. napus MATH* genes were examined using the dual-synteny-plotter function in TBtools (https://github.com/CJ-Chen/TBtools).

### Analysis of Cis-acting regulatory elements in promoter regions

The 2-kilobase (kb) upstream promoter sequences of each *B. napus MATH* gene were extracted from the *B. napus* multi-omics repository (http://yanglab.hzau.edu.cn/). Cis-acting regulatory factors were identified using the public PlantCARE data server (http://bioinformatics.psb.ugent.be/webtools/plantcare/html/). The distribution and functional categorization of the identified elements were subsequently visualized using TBtools.

### Putative miRNA target sites within the *BnMATH* genes

Putative miRNAs targeting *BnMATH* genes were predicted using the psRNATarget server (http://www.zhaolab.org/psRNATarget/analysis?function=3/; accessed February 2, 2025). The analysis was performed using default parameters, except that the expectation threshold was set to 3.0. This stringent threshold was selected because lower expectation scores indicate stronger complementarity between miRNAs and their target transcripts; therefore, miRNA–BnMATH target pairs with expectation scores ≤ 3.0 were retained to reduce false-positive predictions and obtain high-confidence interactions for further analysis. The resulting interaction network between the predicted miRNAs and their corresponding BnMATH transcripts was visualized in R (version 4.2.1) using the ggplot2 and ggalluvial packages.

### Gene expression dynamics of the *BnMATH* genes in *B. napus*

The spatiotemporal expression profiles of the *BnMATH* genes was analyzed in *B. napus* (cultivar ZS11) across various tissues, developmental phases, and in response to hormonal and abiotic stress treatments. Expression data, including FPKM (Fragments per Kilobase of transcript per Million mapped reads) values, were retrieved from the BrassicaEDB repository and the BnIR multi-omics data resource [15, 16]. To facilitate accurate comparison across conditions, FPKM values were log_2_-transformed, ensuring data normalization and reducing the impact of high expression variability. Data visualization and comparative analyses were performed using TBtools, enabling a comprehensive evaluation of the *BnMATH* gene expression patterns and their regulation under different stress and developmental conditions.

### Plant material, growth conditions and treatment

The plant material used in this research was *B. napus L.* Zhongshuang 11 (ZS11), which was bred by the Oil Crop Research Institute of the Chinese Academy of Agricultural Sciences and stored in our laboratory. To investigate the stress-responsive functions of the *MATH* gene family, we performed a qRT-PCR experiment in the *B. napus* cultivar ZS11. The Seeds were germinated in a quarter-strength Hoagland solution and transferred to a half-strength Hoagland solution after seven days under controlled growth conditions (25 ± 1 °C; 14-light/10-h dark photoperiod). Thirty-day-old seedlings were exposed to salt stress (150 mM NaCl), drought stress (20% PEG), or cold stress (4 °C), and the samples were collected at the following time intervals: 0, 1, 3, 6, 12, and 24 h. The samples were flash-frozen in liquid nitrogen and stored at −80 °C.

### RNA extraction and qRT-PCR analysis

Total RNA was isolated from leaf samples using the Vazyme RNAprep Pure Plant Kit. The first strand of cDNA was synthesized using the TRANGEN FastQuant RT Kit according to the manufacturer's protocol. Gene-specific primers for *BnMATH* genes were designed using the NCBI Primer BLAST tool (https://www.ncbi.nlm.gov/tools/primer-blast). The primer design criteria included primer lengths of 18–25 bp, melting temperatures of 58–62 °C, and GC contents of 40–60%, and an expected amplicon size of 100–200 bp. Primer pairs with potential secondary structure, primer dimers, and non-specific amplification were avoided. The selected primer sequences are listed in Supplementary Table S10. qRT-PCR was conducted in a 20 μl reaction volume containing 1 μl of cDNA template, 10 μl of SYBR qPCR Vazyme Master mix, 0.5 μl each primer and 8 μl of nuclease-free water, using Q3-2 Real-time PCR system (Life Technology, USA). Cycling conditions were 95 °C for 2 min, followed by 40 cycles of 95 °C for 15 s and 60 °C for 30 s. Relative expression levels were calculated using the 2^ − ΔΔCT method [17], with the *BnActin* gene used as a normalized control.

### Statistical analysis

Statistical analysis was conducted using IBM SPSS Statistics 22 software (IBM, Armonk, New York, USA). Data are shown as mean ± standard deviation (SD) derived from three independent biological replicates, each with three technical repeats. Statistical significance between groups was assessed with one-way analysis of variance (ANOVA), followed by Fisher’s least significant difference (LSD) post hoc test. The symbol ‘ns’ indicates non-significance, while *, **, ***, and **** represent significance levels at *P* < 0.05, 0.01, 0.001, and 0.0001, respectively.

## Results

### Genome-wide identification of the *MATH* genes in *B. napus*

To identify members of the *MATH* gene family in *B. napus*, a genome-wide search was performed using HMMER 3.0 with the hidden Markov model (HMM) profile of the MATH domain (PF00917). Candidate sequences were further verified using the SMART and HMMSCAN tools to confirm the presence and integrity of the MATH domain. In total, 151 *MATH* genes were identified and named *BnMATH1-BnMATH151* according to their chromosomal positions (Supplementary Table S1).

The physiochemical properties of the identified BnMATH proteins showed considerable variation. The CDS lengths ranged from 366 bp (*BnMATH41*) to 5085 bp (*BnMATH63*), encoding proteins of 121–1694 amino acids. The predicted molecular weight (MW) varied from 13.93 to 190.11 kDa, while theoretical isoelectric points (pI) ranged from 4.45 to 9.65. The aliphatic index ranged from 52.1 to 105.22, and the instability index ranged from 10.32 to 60.93. Most BnMATH proteins were predicted to be hydrophilic, as indicated by negative GRAVY values, except for BnMATH99, which showed a slightly positive GRAVY value of 0.04 (Supplementary Table S1).

### Evolutionary relationships of the *MATH* genes

**Fig. 1:**
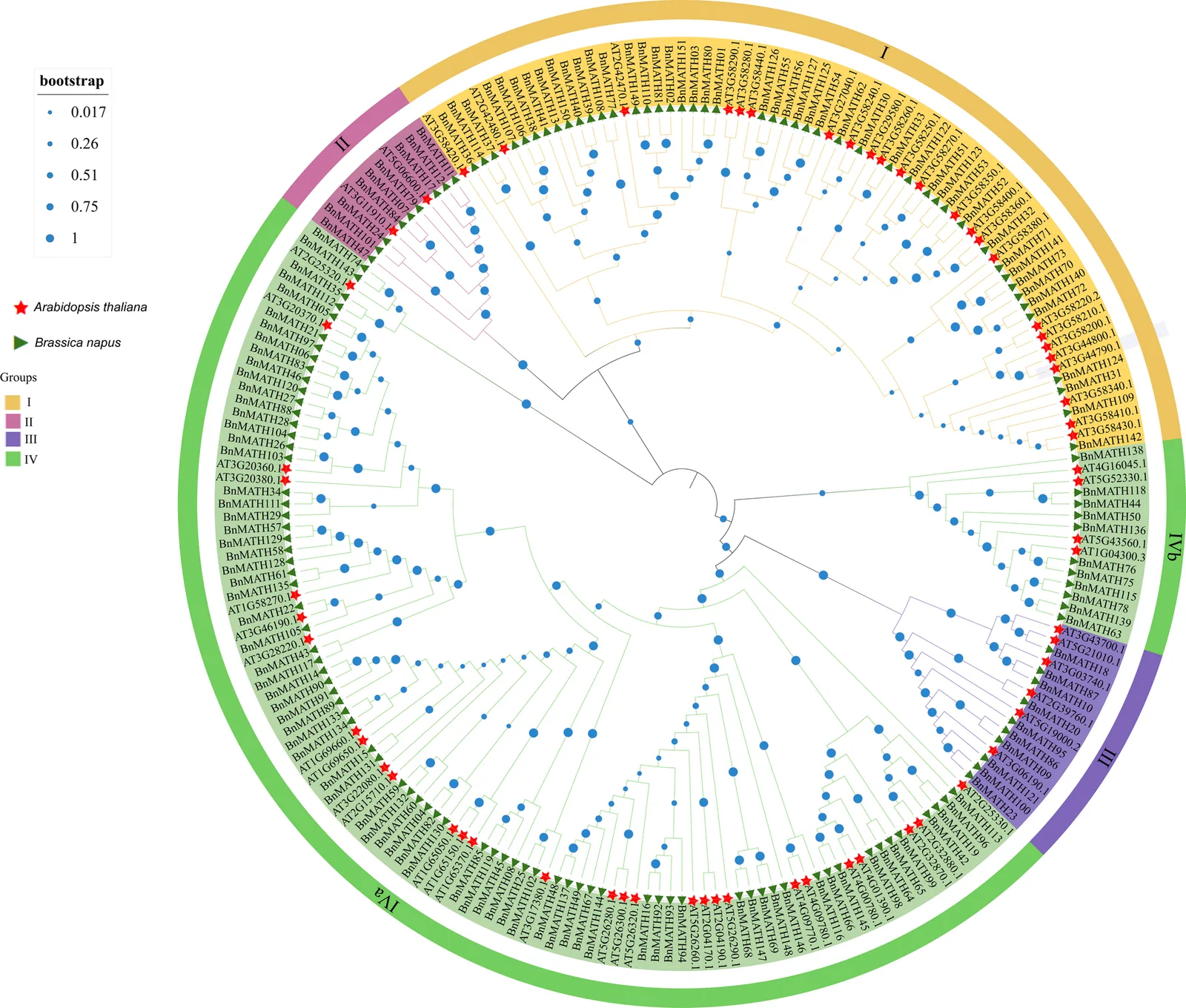
Phylogenetic relationships of MATH proteins from *B. napus* and *A. thaliana*. A total of 216 proteins (151 BnMATH and 65 from AtMATH) were aligned using MUSCLE in MEGA 11, and a neighbor- joining tree was constructed with 1,000 bootstrap replicates. Distinct color clades indicate conserved evolutionary groups and lineage-specific expansion, suggesting potential functional divergence among MATH proteins

An unrooted phylogenetic tree was constructed to analyze the evolutionary relationships of the MATH proteins from *B. napus* and *A. thaliana*. A total of 216 MATH protein sequences were used, including 151 from *B. napus* and 65 from *A. thaliana*. The MATH proteins were classified into four major clades, designated as Groups I-IV (Fig. 1).

Group IV represented the largest clade, containing 119 sequences, and was further divided into two subgroups: Group IVa with 104 members and Group IVb with 15 members. The remaining proteins were distributed in Group I with 70 members, Group II with 11 members, and Group III with 16 members. MATH proteins from *B. napus* and *A. thaliana* were present in all major clades, indicating that the identified BnMATH proteins could be grouped with their *Arabidopsis* homologs.

### Motif patterns gene structure of the *BnMATH* gene family

**Fig. 2:**
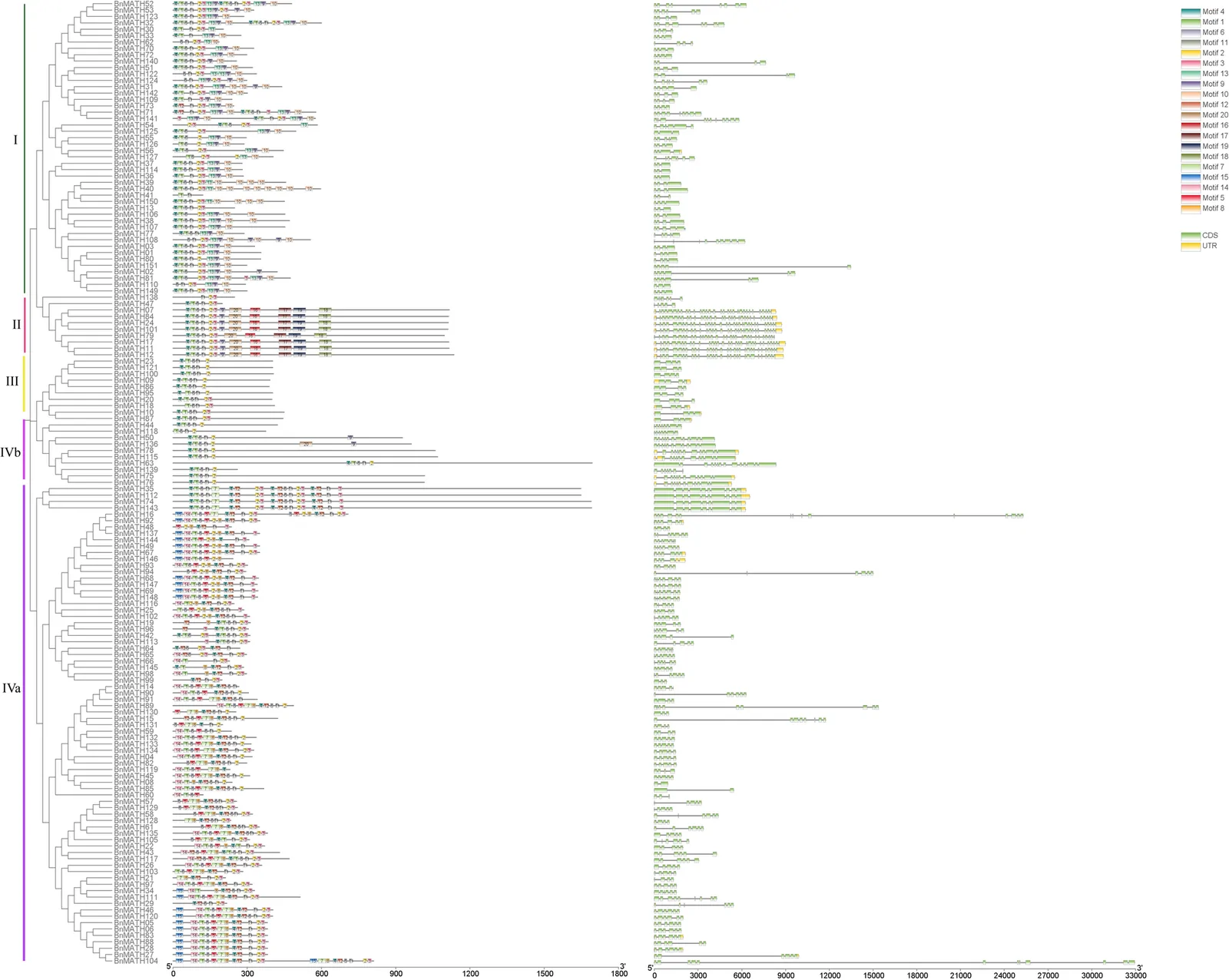
Evolutionary relationships and structural features of *MATH* genes in *B. napus*. **A** Phylogenetic analysis groups BnMATH proteins into distinct clades, indicating evolutionary divergence. **B** Conserved motif patterns reveal structural conservation within clades and potential functional specialization. **C** Gene structure analysis shows exon–intron and untranslated regions (UTRs) variation, supporting both conservation and diversification of the *BnMATH* gene family

The 151 *BnMATH* genes were classified into four major groups, designated as Groups I-IV (Fig. 2A). Group IV was the largest group and was further divided into two subgroups, Group IVa and Group IVb, containing 75 and 11 genes, respectively. Group I contained 46 genes, whereas Groups II and III contained 9 and 10 genes, respectively. Conserved motif analysis using MEME identified 1–20 motifs in each BnMATH protein, with motif lengths ranging from 11 to 50 amino acids (Fig. 2B; Supplementary Table S2). Motif 2, a 15-amino acid conserved motif, was present in most BnMATH proteins but absent in BnMATH14, BnMATH33, BnMATH41, BnMATH60, BnMATH77, BnMATH91, and BnMATH109. Motif 4 was also retained in most groups, while several motifs showed group-specific distribution patterns.

Gene structure analysis based on genomic sequences showed that members within the same subgroup generally had similar exon–intron organizations (Fig. 2C). Overall, the motif composition and gene structure patterns were largely consistent with the phylogenetic grouping of the *BnMATH* gene family.

### Chromosomal localization and collinearity of the *BnMATH* genes

**Fig. 3:**
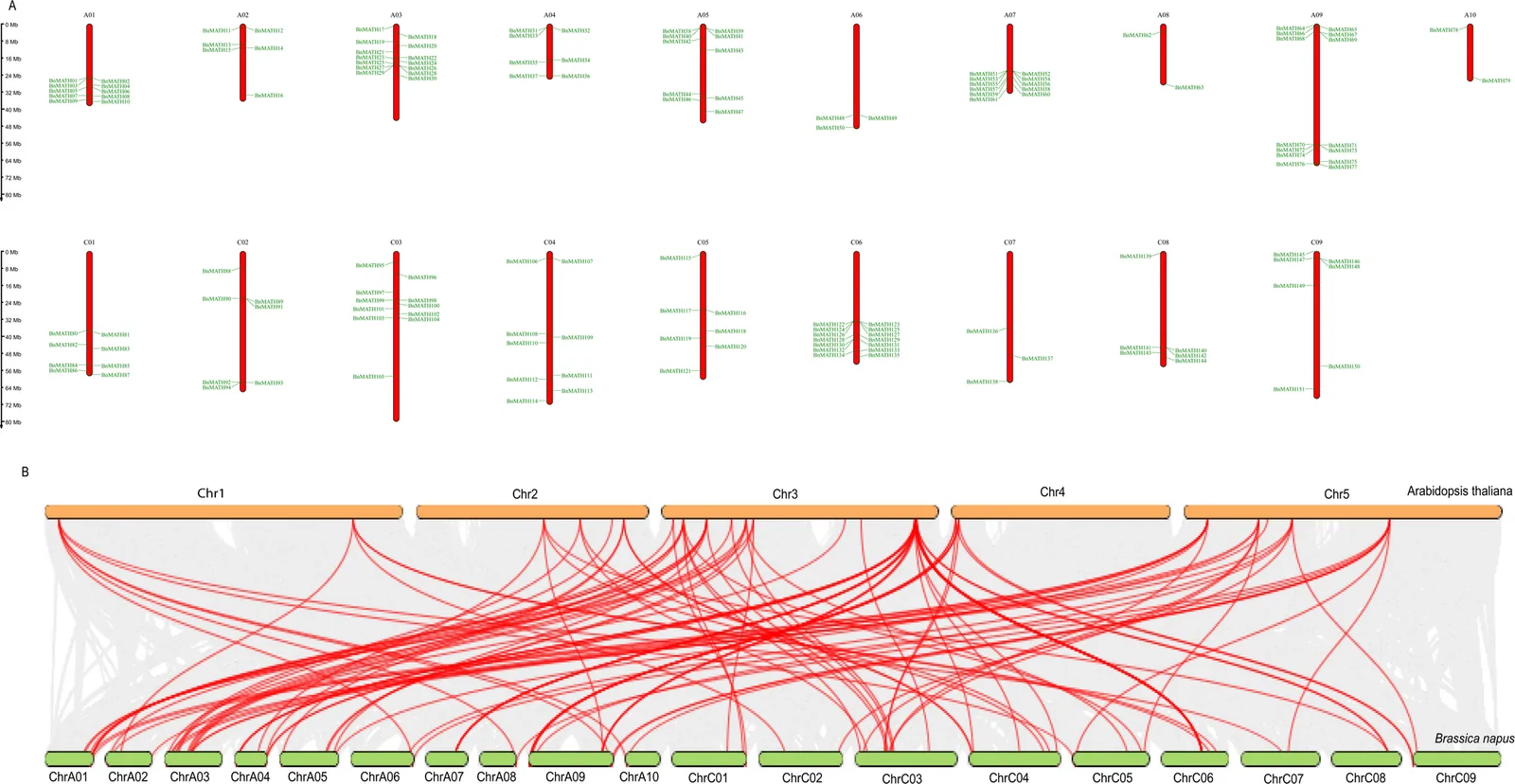
Chromosomal distribution and synteny of *MATH* genes in *B. napus*. **A** *BnMATH* genes are unevenly distributed across the 19 chromosomes, indicating genome-wide expansion. Chromosomes are shown as green bars, with scale in megabases (MB). **B** Synteny analysis with *A. thaliana* reveals conserved genomic blocks (grey) and syntenic gene pairs (red), highlighting evolutionary conservation and duplication-driven diversification of the *MATH* gene family

The 151 *BnMATH* genes were unevenly distributed across 19 chromosomes of *B. napus* (Fig. 3A). Among them, 79 genes were located in the A subgenome, and 72 genes were located in the C subgenome. Chromosomes A03, A09, and C06 contained the highest number of *BnMATH* genes, with 14 genes each, whereas chromosomes A08 and A10 contained the fewest, with two genes each.

**Fig. 4:**
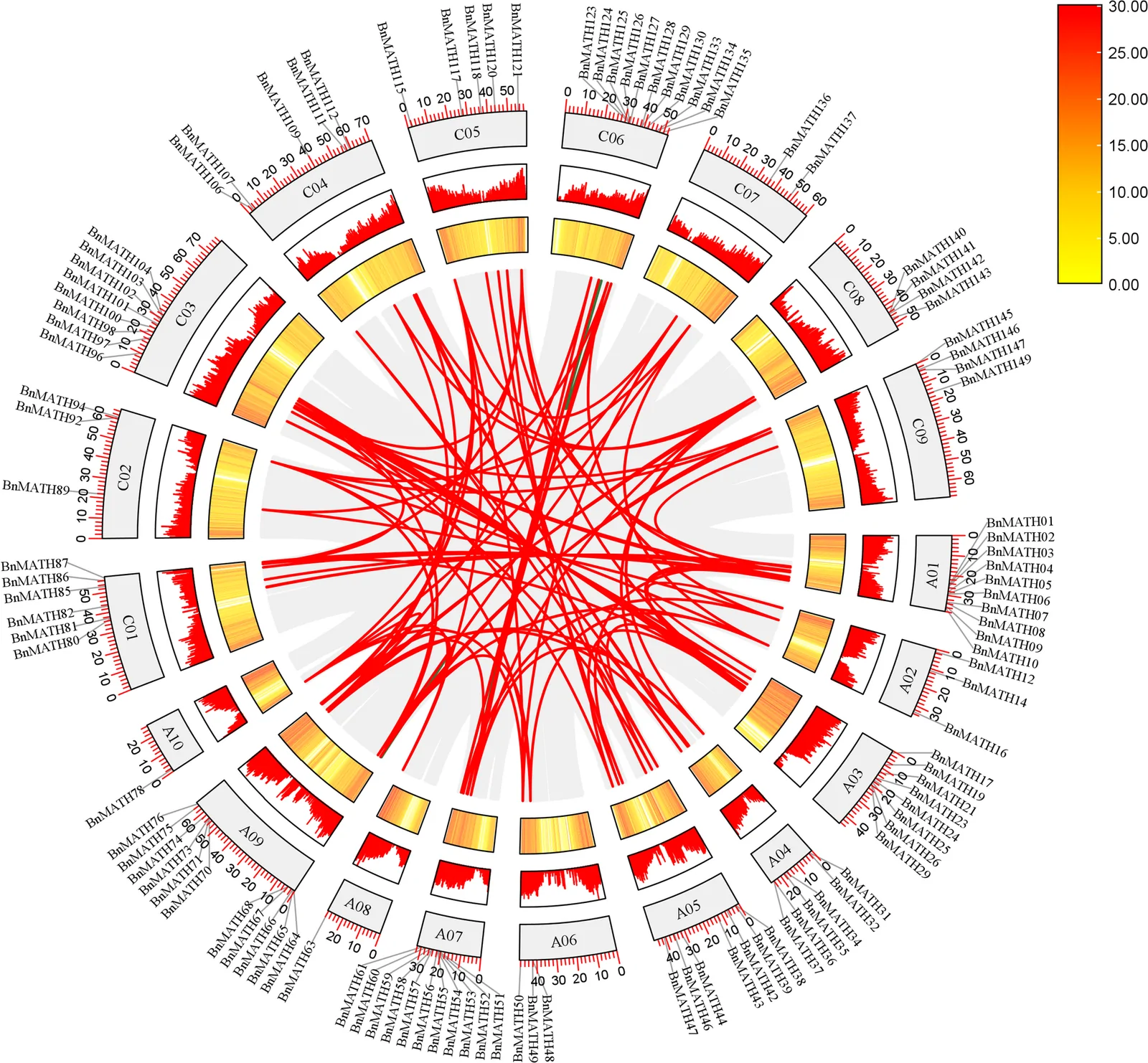
Interchromosomal synteny and duplication patterns of the *BnMATH* genes in *B. napus*. Grey lines represent conserved syntenic blocks, while red and green lines indicate segmental and tandem duplication, respectively, highlighting their key roles in the expansion and diversification of the *BnMATH* gene family

Comparative synteny analysis between *B. napus* and *A. thaliana* identified 79 homologous *MATH* gene pairs (Fig. 3B; Supplementary Table S3). Most syntenic *MATH* genes in *A. thaliana* were located on chromosome 3, while chromosome 4 contained the fewest. Gene duplication analysis identified four tandemly duplicated gene pairs: *BnMATH57/BnMATH61*, *BnMATH64/BnMATH65, BnMATH128/BnMATH135,* and *BnMATH130/BnMATH133*. These tandem duplication events were located on chromosomes A07, A09, and C06, with two pairs located on C06. In addition, 100 segmentally duplicated gene pairs were identified across multiple chromosomes (Supplementary Table S4). Chromosomes A01, A07, A09, and C06 contained the highest numbers of duplicated gene pairs, with 10, 11, 11, and 8 pairs, respectively (Fig. 4).

### miRNA-mediated regulation of the *BnMATH* genes

**Fig. 5:**
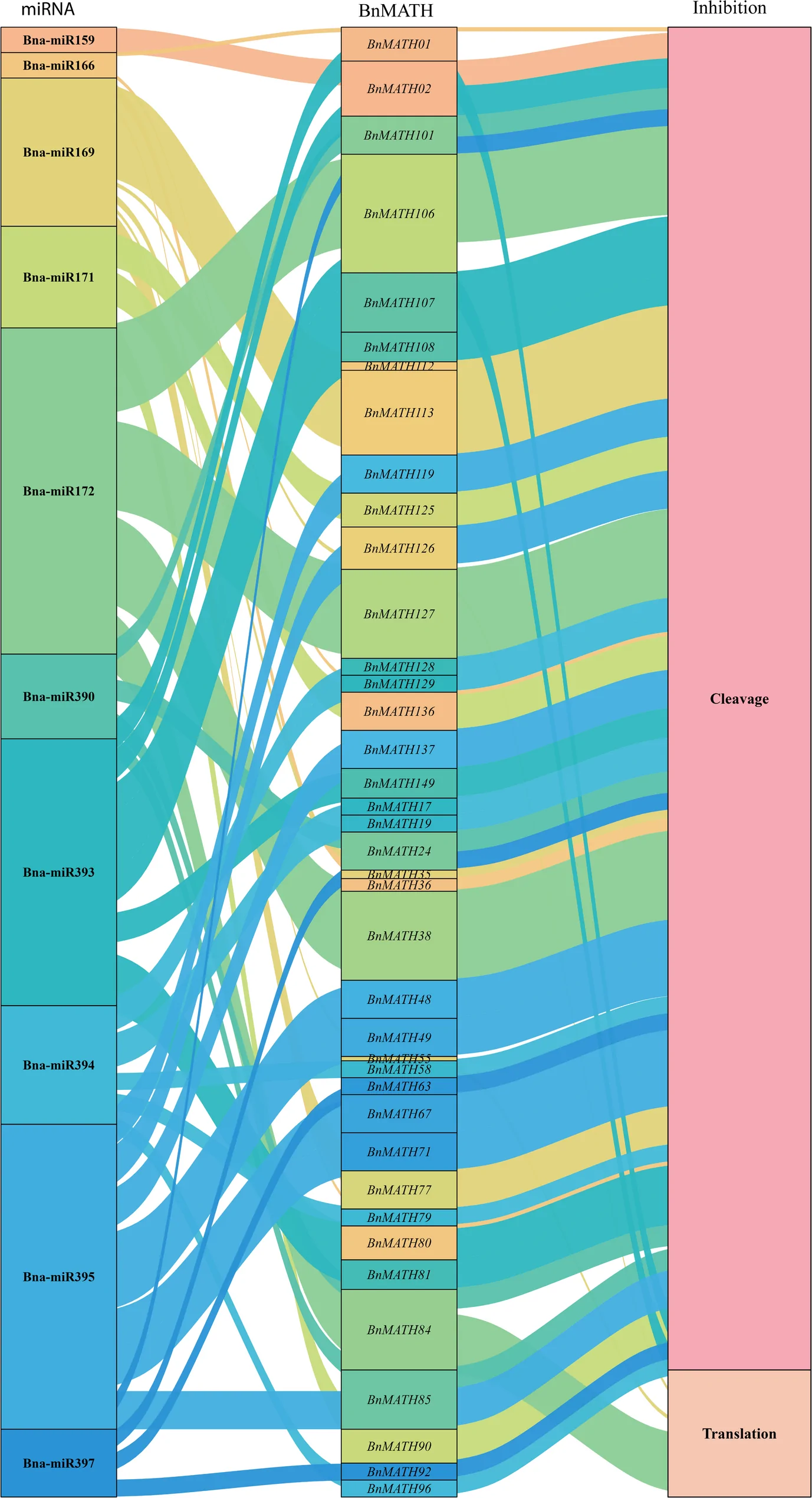
Network representation of predicted miRNA-BnMATH interactions, illustrating putative miRNA targeting sites across the *BnMATH* genes. The network highlights a complex post-transcriptional regulatory framework, suggesting miRNA-mediated control of *BnMATH* gene expression during development and stress responses

MicroRNAs (miRNAs) are well-established regulators of post-transcriptional gene expression and play pivotal roles in plant development and stress adaptation [18]. Prediction of miRNA-target interaction showed that 44 miRNA families potentially targeted 94 *BnMATH* genes in *B. napus* (Supplementary Table 6). Among these, 10 representative miRNA families were selected for visualization including Bna-miR159, Bna-miR166, Bna-miR169, Bna-miR171, Bna-miR172, Bna-miR390, Bna-miR393, Bna-miR394, Bna-miR395, and Bna-miR397 (Fig. 5). Most predicted miRNA-*BnMATH* interactions were classified as target cleavage, whereas a few interactions, including those involving Bna-miR169e and Bna-miR393a-f, were predicted to act through translational inhibition. Several miRNA families, such as Bna-miR169 and Bna-miR171, Bna-miR172, and Bna-miR393, were predicted to target multiple BnMATH genes, including *BnMATH77*, *BnMATH90, BnMATH113*, *BnMATH125*, and *BnMATH136*.

### Cis-regulatory elements in the *BnMATH* gene promoters

**Fig. 6:**
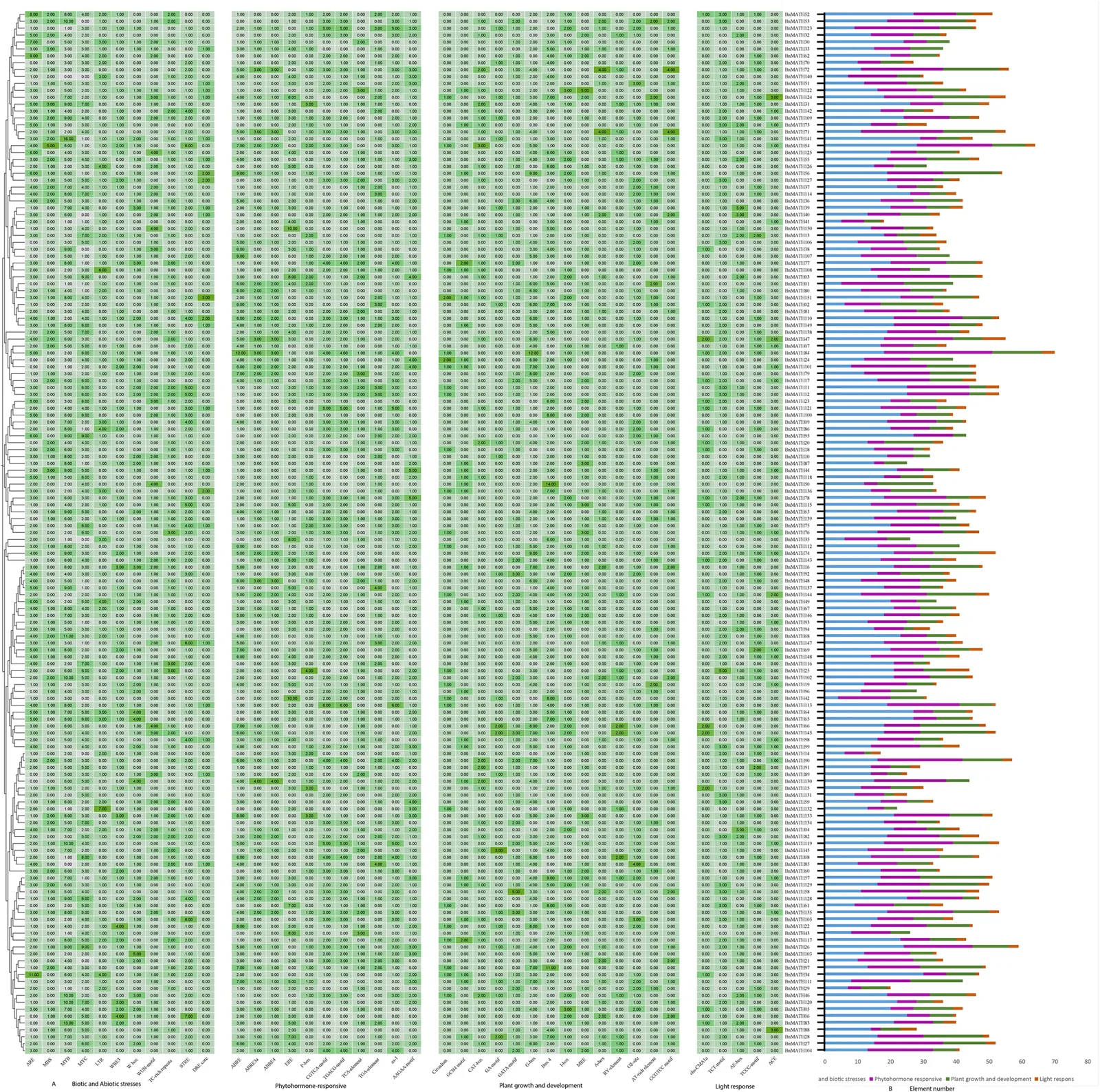
Cis-regulatory architecture of the *MATH* gene family in *B. napus*. **A** Distribution of cis-acting promoter elements across the *BnMATH* genes, with colors representing distinct regulatory classes. **B** Quantitative categorization of cis-elements into major functional groups, highlighting the regulatory complexity and potential involvement of *BnMATH* genes in stress-hormone- responsive pathways

Cis-regulatory elements play a central role in controlling gene expression during development, hormone signalling, and stress responses [19]. The 2-kb upstream regions of the 151 *BnMATH* genes were analyzed using PlantCARE to identify cis-regulatory elements. A total of 6,175 cis-elements were identified and classified into four functional groups: stress-responsive (11 types), phytohormone-responsive (11 types), growth- and development-related (14 types), and light-responsive (5 types; Supplementary Table S5). The major cis-elements included stress-related MYB, MYC, and ARE elements; hormone-responsive ABRE and ERE; growth-associated G-box and Box4; and the light-responsive TCT motif were most abundant (Fig. 6A, B).

### Analysis of expression patterns of the *BnMATH* genes

**Fig. 7:**
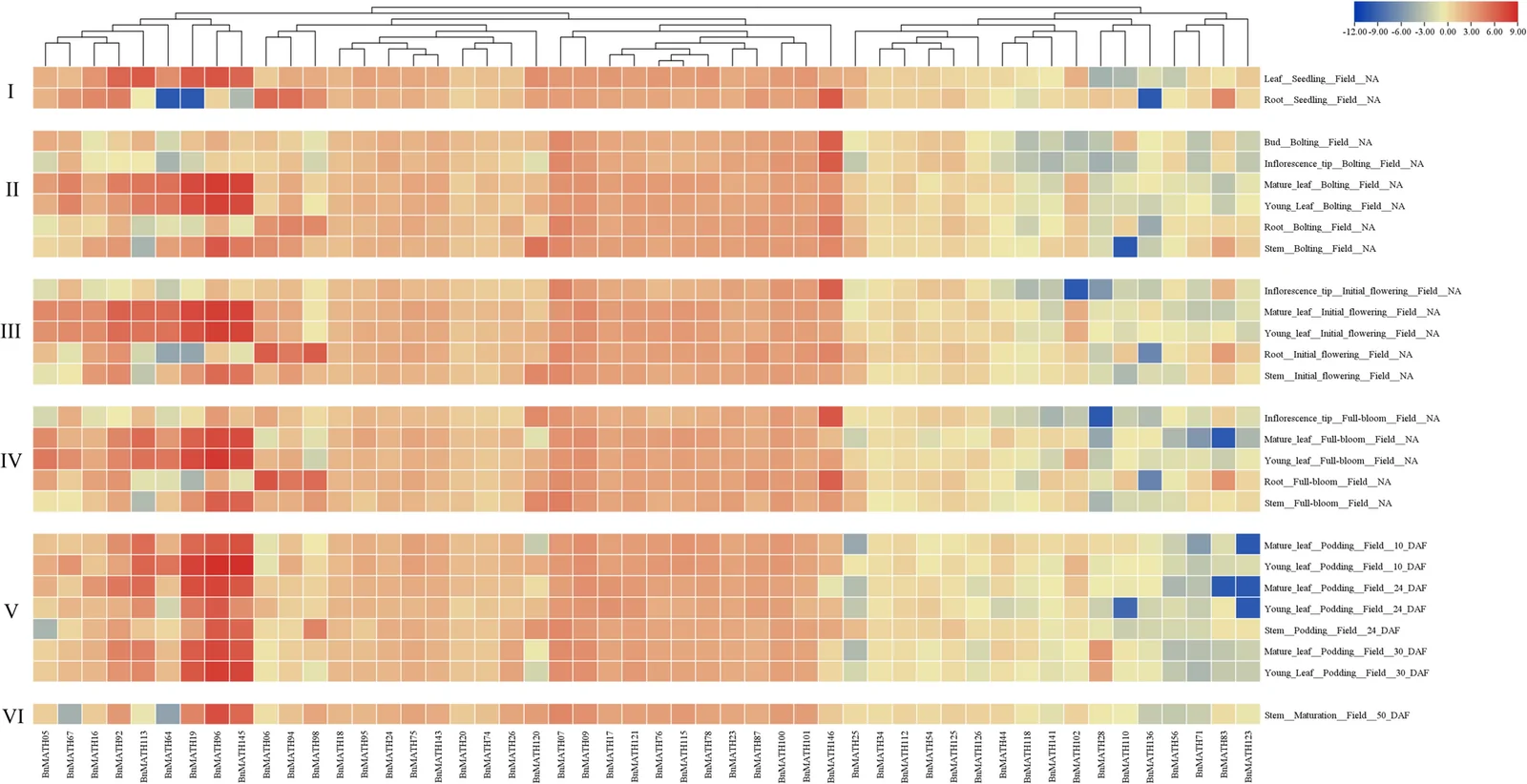
Developmental expression patterns of *BnMATH* genes in *B. napus* cultivar ZS11 across six stages: I (seedling), II (bolting), III (initial flowering), IV (full flowering), V (podding), and VI (maturation). FPKM values were used for heatmap generation with log2 normalization. High and low levels of expression are respectively indicated in red and blue

To evaluate the developmental expression patterns of *BnMATH* genes, transcriptome data for 50 *BnMATH* genes were retrieved from BrassicaEDB [15] and analyzed across six developmental stages I-VI: seedling, bolting, early flowering, full blooming, podding, and ripening. Expression profiles were also evaluated in different tissues, including roots, stems, leaves, floral buds, and inflorescence apex (Fig. 7; Supplementary Table S7). Among the analyzed genes, *BnMATH96* and *BnMATH145* showed consistently high expression across multiple tissues. *BnMATH19* displayed broad expression but relatively lower expression in roots, while *BnMATH146* showed higher expression in roots and inflorescences. Several genes, including *BnMATH83*, *BnMATH56, BnMATH25, BnMATH26,* and *BnMATH102*, showed stage- or tissue-specific expression patterns.

**Fig. 8:**
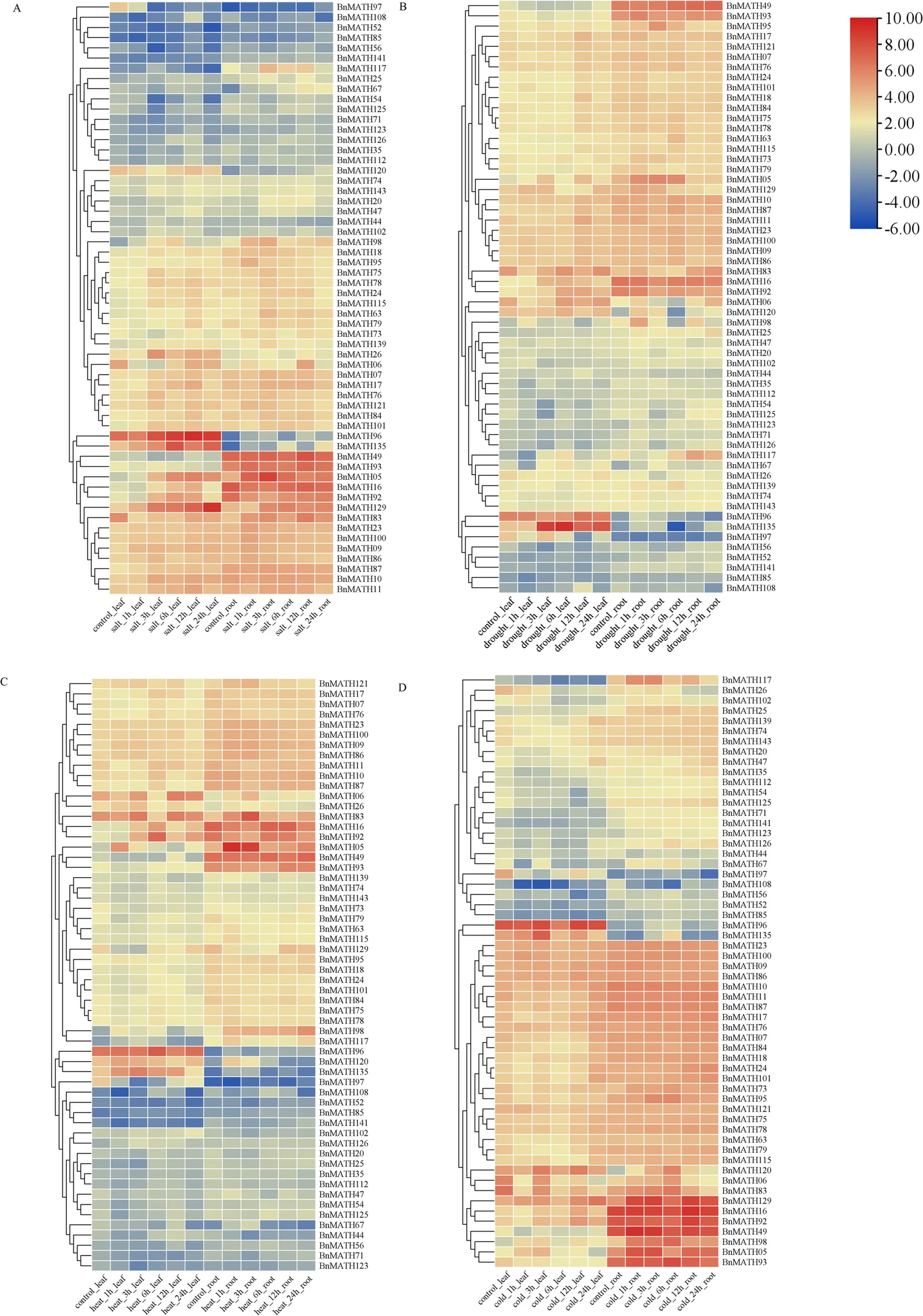
Transcriptional dynamics of the *B. napus MATH* gene family under abiotic stress. Heatmaps depict differential expression patterns in leaf and root tissues across successive time points in response to salt (**A**), drought (**B**), heat (**C**), and cold (**D**) stress. Expression levels are presented as log2 (FPKM). Distinct tissue-and stress-specific patterns highlight dynamic regulation of *BnMATH* genes, suggesting their role in stress signalling and adaptive responses

**Fig. 9:**
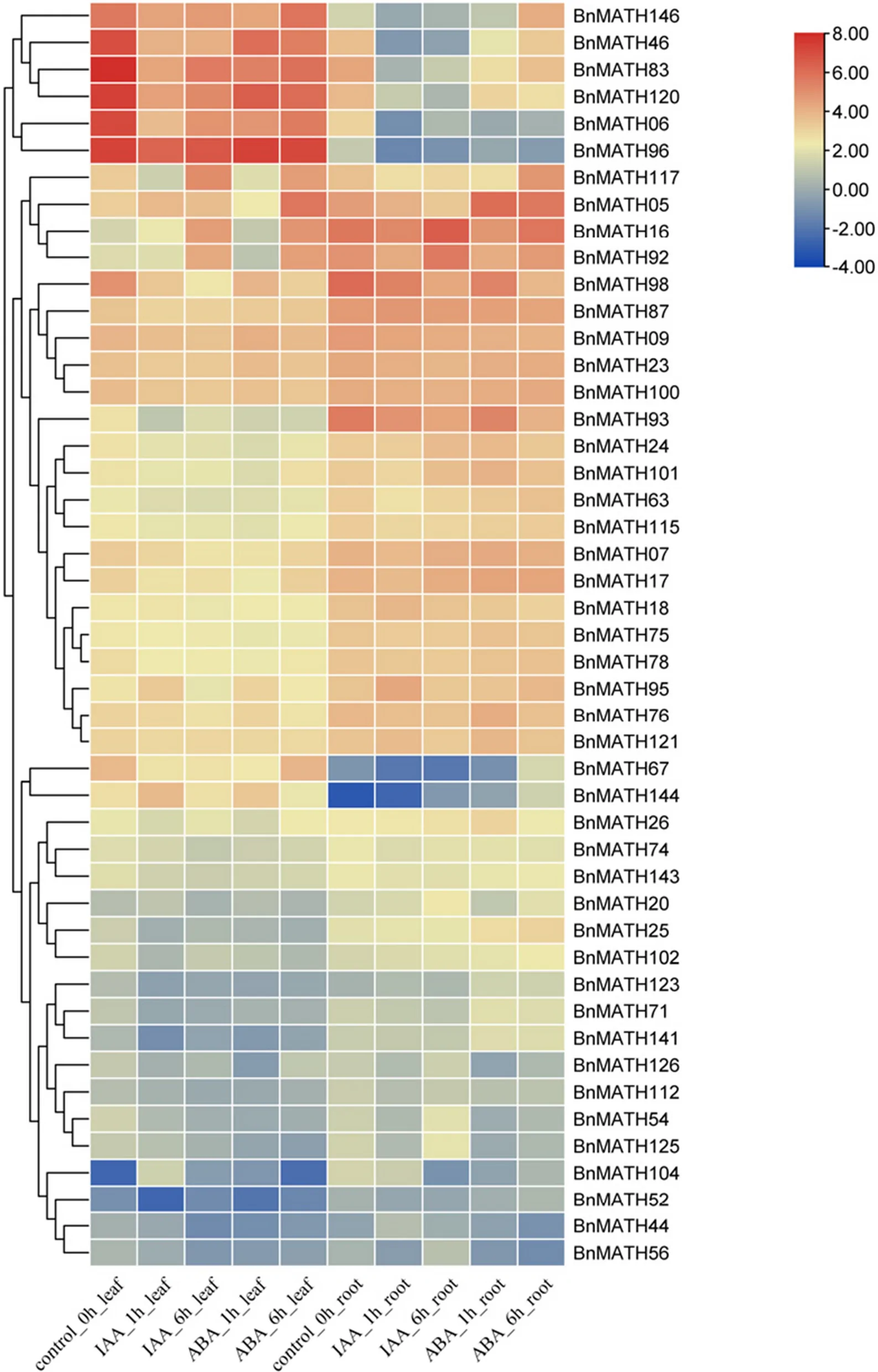
Heatmap of *MATH* gene expression in *B. napus* in response to IAA and ABA in leaf and root tissues across different time points. Expression levels are presented as log2 (FPKM). Distinct tissue-and time- dependent patterns highlight hormone-responsive regulation, suggesting the role of *BnMATH* genes in IAA and ABA-mediated signalling pathways

To evaluate the expression of *BnMATH* genes under abiotic stress and hormone treatments, transcriptome data were retrieved from the BnIR database [16] and analyzed at multiple time points (Figs. 8 and 9; Supplementary Tables S8 and S9). Under salt stress, *BnMATH129, BnMATH96, BnMATH135*, and *BnMATH26* were upregulated in leaves, whereas *BnMATH97* and *BnMATH54* were downregulated. In roots, *BnMATH05, BnMATH06*, and *BnMATH98* were upregulated, while *BnMATH108* was downregulated (Fig. 8A). Under drought stress, *BnMATH135* and *BnMATH05* were upregulated in leaves, and *BnMATH117* was upregulated in roots, whereas *BnMATH97* was downregulated (Fig. 8B). Heat stress induced the expression of *BnMATH83*, *BnMATH05*, and *BnMATH98* in roots (Fig. 8C). Under cold stress, *BnMATH135* was upregulated in leaves, while *BnMATH129, BnMATH98, BnMATH120*, and *BnMATH117* were upregulated in roots. In contrast, *BnMATH97, BnMATH108,* and *BnMATH26* were downregulated (Fig. 8D).

Hormone treatments also showed distinct expression patterns. Under IAA treatment, *BnMATH16* and *BnMATH92* were upregulated in leaves, whereas *BnMATH06, BnMATH96, BnMATH83*, and *BnMATH120* were downregulated in both leaves and roots. Under ABA treatment, *BnMATH06, BnMATH83, BnMATH120, BnMATH46*, *BnMATH05, BnMATH16* and *BnMATH92* were upregulated in leaves, while *BnMATH06, BnMATH96, BnMATH67,* and *BnMATH144* were downregulated in roots (Fig. 9). Overall, BnMATH genes showed tissue-, stage-, and hormone-dependent expression patterns.

### *BnMATH* qRT-PCR expression profile under abiotic conditions

**Fig. 10:**
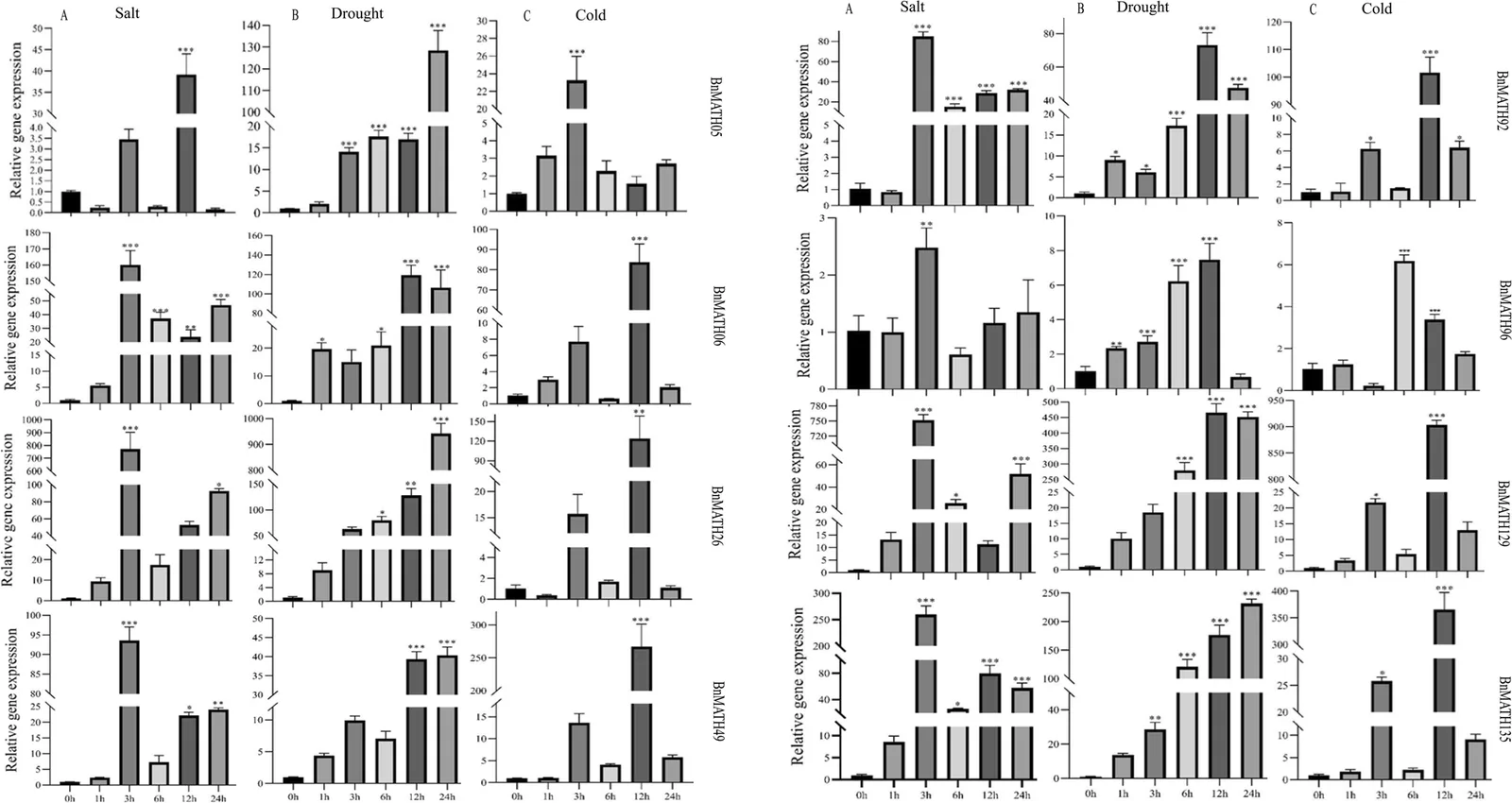
Expression analysis of *BnMATH* genes in *B. napus* under abiotic stresses. **A**. salt, **B**. drought and **C**. cold treatments at different time intervals (0, 1, 3, 6, 12, and 24 h). Data are presented as mean ± SD (*n* = 3 biological replicates). Statistical significance was determined by one-way ANOVA followed by the LSD multiple comparisons test. The ‘ns’ indicates non-significant differences, while *,**, ***, and **** represent 11. *P* < 0.05, 0.01, 0.001, and 0.0001, respectively. The dynamic expression patterns indicate stress- specific induction of *BnMATH* genes, supporting their role in abiotic stress responses

qRT-PCR was performed to observe the temporal expression patterns of eight *BnMATH* genes (*BnMATH05, BnMATH06, BnMATH26, BnMATH49, BnMATH92, BnMATH96, BnMATH129* and *BnMATH135*) in *B. napus* under salt, drought, and cold stress conditions for 1, 3, 6, 12, and 24 h (Fig. 10A-C).

Under salt stress, seven genes (*BnMATH06, BnMATH26, BnMATH49, BnMATH92, BnMATH96, BnMATH129,* and *BnMATH135*) showed increased transcript level at 3 h, with approximately 160, 750, 95, 80, 2.5, 750 and 250-fold changes relative to the control, respectively. *BnMATH05* was also upregulated under salt stress, reaching its highest expression level of approximately 40-fold at 12 h (Fig. 10A).

Under drought stress, all eight *BnMATH* genes showed increased transcript levels at different points. *BnMATH05, BnMATH26, BnMATH49, BnMATH129,* and *BnMATH135* showed their highest expression at 24 h with approximately 130-, 930-, 230-, 180-, and 210- fold increases relative to the control, respectively. In contrast, *BnMATH06*, *BnMATH92*, and *BnMATH96* reached peak expression at 12 h and declined at 24 h (Fig. 10B).

Under cold stress, most *BnMATH* genes showed moderate changes in transcript abundance. *BnMATH06, BnMATH26, BnMATH49, BnMATH92, BnMATH129*, and *BnMATH135* reached maximum expression at approximately 12 h, followed by a decline. *BnMATH05* and *BnMATH96* showed earlier expression peaks at 3 and 6 h, respectively (Fig. 10C). Overall, the dynamic, stress-responsive expression patterns of the *BnMATH* genes highlight their role in regulating critical molecular mechanisms for abiotic stress tolerance.

## Discussion

### Polyploidization-driven expansion and diversification of the *MATH* gene family in *B. napus*

*B. napus*, an allotetraploid crop derived from the natural hybridization of *B. rapa* and *B. oleracea*, is a primary source of edible oil and a significant contributor to biofuel production. Its high-yield potential, rich oil content and broad environmental adaptability make it one of the most widely cultivated species [1]. In China, *B. napus* remains a major agricultural commodity, but its productivity is under increasing pressure from abiotic stresses, including heat, drought, cold, salinity, and heavy metal toxicity. These stresses collectively impair seed development and threaten yield stability [20].

The availability of the *B. napus* reference genome has enabled a detailed investigation of the gene families involved in growth, development, and stress adaptation [11, 21–23]. *MATH* genes, which are essential components of ubiquitin E3 ligases, play a key role in mediating plant development, hormone signalling, immunity, and environmental stress tolerance [4]. The *MATH* genes have been catalogued in various plant species, including *A. thaliana*, *B. rapa*, rice and maize [9, 10, 24, 25]. Despite its global economic significance, *B. napus* lacks identification and characterization of the *MATH* gene family.

This study identified 151 *MATH* genes in *B. napus,* representing the most comprehensive catalog of this gene family reported to date in plants. This number substantially exceeds those reported in related species, including *B. rapa* (90), *A. thaliana* (77), rice (75) and the *Solanaceae* family (58) [4, 9, 11], highlighting a lineage-specific expansion in *B. napus*. The wide variation in protein length, molecular weight, PI, and stability suggests possible functional diversification among BnMATH proteins. This expansion is a direct consequence of *B. napus'* amphidiploid origin. It arose from the hybridization of *B. rapa* and *B. oleracea*, followed by whole-genome duplication and extensive gene retention. Polyploidization increased gene copy number and facilitated segmental and tandem duplications, providing opportunities for the functional diversification within the *MATH* gene family. Many retained *BnMATH* genes exhibit stress- and hormone-responsive expression patterns, suggesting that they were retained due to their adaptive advantages under fluctuating environmental conditions. Together, these findings reflect the polyploid evolutionary history of *B. napus* and its ecological and agronomic requirements for a robust regulatory network that supports stress tolerance, growth, and developmental plasticity.

### Comparative phylogenomic discovery, evolutionary retention and functional modification of the MATH proteins

Phylogenetic analysis grouped BnMATH proteins into four main clades. Group IV showed the most pronounced expansion, reflecting lineage-specific amplification. The comparative inclusion of *A. thaliana* sequences identified deeply conserved clades shared across dicots, suggesting an ancient origin of the *MATH* gene family followed by a species-level diversification. Consistent with earlier classifications, *MATH* genes were classified into four subclasses: class I proteins include the BTB/POZ domain, which is involved in transcription factor turnover during development and stress [4, 26, 27]; class II MATH-UBP proteins perform the function of deubiquitinating enzymes in ubiquitin-dependent proteolysis; class III MATH- filament and MATH PEARLI-4 proteins are less characterized but have been linked to chloroplast function and phospholipase-like signalling, and class IV TRAF/MATH-only proteins mediating protein–protein interactions [24, 28].

### Gene architecture, motif variation, chromosomal dynamics, and syntenic expansion of the *BnMATH* gene family in the *B. napus*

The *BnMATH* genes within each group exhibited conserved domain organization and motif composition, supporting their shared evolutionary history. Group IV exhibited the greatest expansion and comprises two subgroups (IVa and IVb), which displayed diversification and lineage-specific amplification. Motif analysis revealed that nearly all BnMATH proteins contain motif 2, which is highly conserved and has been identified as an essential structural signature for TRAF/MATH integrity in *A. thaliana* [4, 28]. Group- or subgroup-specific motifs, such as motif 4, mark the site of functional divergence. The similar motif composition and exon–intron organization among members within the same phylogenetic subgroup suggest evolutionary conservation of the *BnMATH* gene family. In contrast, group-specific motif distribution, particularly variation in terminal regions, may reflect structural diversification and potential functional differentiation among BnMATH proteins. Gene structure analysis confirmed these relationships, as members within each group showed similar exon–intron architectures. Chromosomal localization revealed a random distribution of *BnMATH* genes across the 19 chromosomes, with many regions acting as duplication hotspots [10]. Analysis of Synteny and collinearity identified extensive segmental duplication events between the A and C subgenomes, consistent with polyploid ancestry. Tandem duplications contributed to further expansion. The presence of both tandem and segmental duplication events suggests that gene duplication contributed to the expansion of the *BnMATH* gene family in *B. napus*. The large number of segmentally duplicated gene pairs indicates that segmental duplication may have played a more prominent role than tandem duplication in the diversification of this gene family. Overall, gene expansion reflects the combined effects of whole-genome duplication, segmental amplification, and gene retention. These effects promote both functional redundancy and novel diversification. *BnMATH* gene pairs with similar expression profiles likely retain redundant functions, while those with divergent expression patterns likely underwent functional diversification following polyploidization. The integration of conserved motifs, stable gene architecture, and chromosomal collinearity, however, demonstrated that the *BnMATH* gene family exhibits stable evolutionary conservation and innovation. This supports the complex regulatory pathways required for growth and stress adaptation.

### Promoter structure highlights the multifaceted regulatory roles of the *BnMATH* genes under environmental stress

About 6,000 cis-elements were found across 151 genes, reflecting remarkable regulatory diversity and notable enrichment in groups responsive to stress, hormone, light and development. The abundance of stress-, hormone-, developmental-, and light-responsive cis elements in the promoter regions suggests that *BnMATH* genes may be transcriptionally regulated by multiple environmental and developmental signals. The most abundant elements were MYB, MYC and ARE, establishing strong associations between *BnMATH* gene promoters and networks responsive to abiotic stress. MYB and MYC transcription factors mediate broad transcriptional reprogramming under osmotic stress conditions. ARE motifs contribute to redox homeostasis, consistent with recent reports of stress-inducible promoters in rapeseed [29, 30]. Additionally, the enrichment of ABRE and ERE motifs links *BnMATH* genes to ABA- and ethylene-responsive transcriptional networks. Previous studies have shown that ABA mediates drought stress adaptation and that ethylene regulates waterlogging tolerance. This dual enrichment suggests that *BnMATH* genes are involved in orchestrating hormonal crosstalk during stress adaptation [31]. Beyond stress-related regulation, the frequent presence of G-box and Box4 elements indicates that *BnMATH* gene promoters are involved in light-mediated transcriptional control. These elements recruit bZIP and BBX transcription factors, which have been shown to regulate seed development under light-dependent conditions in rapeseed [32]. These findings suggest that *BnMATH* genes may act as integrative regulators, linking environmental signals, hormone-mediated pathways, and developmental processes. Notably, the enrichment of stress- and hormone-responsive cis-elements aligns with the transcriptional induction of *BnMATH* genes in response to abiotic stress and hormone treatments. This suggests that these promoter features directly contribute to their stress- and hormone-responsive expression patterns. Further experimental validation through promoter-reporter assays is essential to verify these regulatory interactions and define the functional roles of the individual cis-elements.

### MicroRNAs-MATH interaction orchestrates post-transcriptional control of the *MATH* gene family in the *B. napus*

MicroRNAs (miRNAs) are non-coding RNAs composed of 21 nucleotides that regulate gene expression after transcription by integrating developmental and environmental signals [33, 34]. A total of 44 distinct families were predicted to target the *MATH* genes in *B. napus* (Supplementary Table S6), indicating the presence of a highly interconnected, small RNA-mediated regulatory network. The major conserved families, including miR159, miR169, miR172, miR390, miR393, miR394, miR395, and miR396, showed the strongest potential for interaction, suggesting multilayered post-transcriptional regulation (Fig. 7).

The predicted regulation of multiple *BnMATH* genes by different miRNA families suggests that post-transcriptional regulation may contribute to the functional modulation of the *BnMATH* gene family. The presence of both cleavage- and translational inhibition-based interactions indicates that BnMATH genes may be regulated through different miRNA-mediated mechanisms. While most predicted miRNA-BnMATH interactions involve mRNA cleavage, a smaller subset, including miR196e and miR393a-f, may act through translational inhibition. This reflects the diversity of mechanisms by which miRNAs exert control. Several conserved families are associated with adaptation to abiotic stress. For example, miR169 is a key regulator of the NF-YA transcription factor in drought signalling [18]. Similarly, miR395, which controls sulfur assimilation and has been predicted to interact with a *BnMATH* gene, suggests potential coordination between sulfur metabolism and ubiquitin-related signalling. Previous research on the *B. napus* variety oleifera has shown that bna-miR160, bna-miR164b, bna-miR394a-c, and bna-miR395a are triggered by sulfate deficiency and cadmium exposure. BnASP1, 3, and 4 have been identified as the targets of miR395. Furthermore, bna-miR164a, bna-miR5712, and bna-miR6029 have been associated with responses to cold stress and lipid metabolism. These findings underscore the multifaceted roles of miRNAs in regulating energy balance, maintaining membrane integrity, and enhancing stress tolerance [35].

Importantly, to strengthen the biological relevance of the predicted miRNA-Bn MATH interaction network, several qRT-PCR validated *BnMATH* genes were found to coincide with predicted miRNA targets. Specifically, *BnMATH49*, *BnMATH92*, *BnMATH96*, and *BnMATH129* displayed dynamic, stress-responsive expression patterns in qRT-PCR analyses and were predicted targets of Bna-miR395, Bna-miR397, and Bna-miR394, respectively. The overlap between the experimentally validated genes and the predicted miRNA targets supports the regulatory significance of the miRNA-BnMATH network. It also suggests that miRNA-mediated post-transcriptional control may fine-tune *BnMATH* gene expression during abiotic stress responses.

### Functional roles of the MATH protein in developmental regulation and stress adaptation

Most genes exhibited consistent expression patterns between qRT-PCR and RNA-seq data. Specifically, *BnMATH05* showed similar expression trends under cold stress; *BnMATH06,* under drought stress; *BnMATH26,* under salt stress; and *BnMATH49,* under cold stress. Additionally, *BnMATH129* exhibited a similar expression profile under both drought and cold stress, and *BnMATH135* displayed consistent expression under both salt and cold conditions.

Under salt stress, several *BnMATH* genes, including *BnMATH06*, *BnMATH26*, *BnMATH129*, and *BnMATH13,* showed rapid and pronounced induction at early time points (1–3 h), suggesting their potential involvement in the initial perception and transmission of stress signals. In contrast, *BnMATH05* exhibited delayed but sustained induction, with peak expression at later stages, suggesting a role in downstream stress acclimation and cellular adjustment. Similar temporal stratification occurred under drought stress. The Continuous time-dependent upregulation of the *BnMATH05*, *BnMATH26*, *BnMATH49*, *BnMATH129*, and *BnMATH135* genes reflected their roles in long-term drought adaptation. In contrast, *BnMATH06, BnMATH92*, and *BnMATH96* displayed early but transient induction, consistent with roles in early or intermediate stress signalling. Cold stress elicited comparatively transient and moderate transcriptional responses. Several genes peaked at intermediate time points, followed by repression. This underscores the stress-specific regulatory dynamics within the *BnMATH* family.

The stress-responsive expression patterns of the selected *BnMATH* genes indicate that different members may participate in distinct phases of abiotic stress responses. The strong early induction of several genes under salt stress suggests rapid transcriptional activation, whereas the sustained induction of some genes under drought stress may reflect their possible involvement in prolonged stress responses. These findings support the potential role of *BnMATH* genes in abiotic stress regulation in *B. napus*.

These expression patterns align well with the established role of the MATH proteins in other plant species. In *A. thaliana*, the deubiquitylating enzymes UBP12 and UBP13 regulate leaf growth, root meristem activity, and flowering time by stabilizing key developmental regulators [36, 37]. Members of the BPM subfamily act as adapters for CUL3-based E3 ubiquitin ligases, directing transcription factors, such as DREB2A and RAP2.4, toward proteasomal degradation. This prevents excessive stress signalling and maintains the balance between growth and stress [27, 38, 39]. Similarly, only MATH domain-containing proteins, such as *AtTRAF1a* and *AtTRAF1b*, participate in immune regulation and autophagy under nutrient limitation [40–42]. In crop species, including maize and wheat, *ZnMAB1* and *TaMAB1/2* play key roles in reproductive development, regulating germline cell division and early embryogenesis [25, 43].

*BnMATH* genes displayed highly specific spatial expression patterns across different tissues. This pattern suggests that they are involved in plant growth and reproductive development. The differential regulation of these genes in leaves and roots under multiple abiotic stresses mirrors the regulatory behaviour of *A. thaliana* BPM proteins. These proteins fine-tune stress-responsive transcription factors, balancing defence and development. The strong transcriptional responsiveness of *BnMATH* genes to ABA suggests their integration into hormone-mediated stress signalling pathways. Auxin-induced expression links specific *BnMATH* genes to developmental regulation. Therefore, the combined transcriptome and qRT-PCR evidence supports a model in which *BnMATH* genes participate in both early stress signalling and later adaptive processes through ubiquitin-mediated regulatory mechanisms.

Despite these strong expression-based and comparative functional insights, it is important to note that direct functional validation of *BnMATH* genes through genetic or molecular approaches is lacking. Consequently, while the present data provide a robust framework for functional inference, targeted experimental validation is essential to confirm the precise molecular roles of individual *BnMATH* genes in stress tolerance and development.

### Future strategies

Future studies should focus on the functional validation of *BnMATH* genes through targeted genome-editing strategies, including CRISPR/Cas9-mediated mutagenesis and complementary overexpression approaches. These methods will provide critical insights into the roles of *BnMATH* genes in plant development and stress adaptation. In parallel, degradome sequencing and transient expression assays can be employed to validate miRNA-target interactions experimentally. Furthermore, promoter-reporter analyses will be essential for understanding the regulatory role of stress- and hormone-responsive cis-elements in controlling *BnMATH* gene expression.

## Conclusion

We present the first comprehensive, genome-wide identification and systematic characterization of the *MATH* gene family in *B. napus*. A total of 151 *BnMATH* genes were identified, revealing substantial evolutionary expansion driven by extensive segmental and tandem duplication events. The presence of conserved motifs, patterns, and stable exon–intron structures, as well as strong syntenic relationships with *Arabidopsis,* suggests deep evolutionary conservation. Meanwhile, promoter and miRNA analyses highlight a multilayered regulatory landscape. Transcriptome profiling identified several stress-responsive candidate genes, including *BnMATH05*, *BnMATH06*, *BnMATH26*, *BnMATH49*, *BnMATH92*, *BnMATH96*, *BnMATH129*, and *BnMATH135*. These were validated using qRT-PCR. These genes exhibited strong and dynamic induction under salt and drought stress, suggesting that they play pivotal roles in early signalling and sustained stress adaptation. These findings provide valuable insights into the evolutionary and functional attributes of the *BnMATH* gene family and establish a solid foundation for future mechanistic studies aimed at enhancing stress tolerance in *B. napus*.

## Supplementary Information

Supplementary Material 1.

## Data Availability

The datasets supporting the findings of this study are fully available within the article and its supplementary materials.

## Abbreviations

MATH: Meprin and TRAF-C homology
HMM: Hidden Markov Model
miRNA: Micro-RNA
qRT-PCR: Real-Time Quantitative Transcription PCR
TRAF: Tumor necrosis factor
BTB: Broad-complex, tramtrack, and bric-à-brac
POZ: Poxvirus and zinc finger
UBP: Ubiquitin-specific protease
RING: Really interesting new gene
*BnMATH*: *Brassica napus MATH* Genes
*B. napus*: *Brassica napus*
Pl: Isoelectric points
GRAVY: Grand Average of Hydropathicity
NJ: Neighbor-joining
CDS: Coding sequences
GFF3: Genome annotation
kb: Kilobase
ANOVA: Analysis of variance
LSD: Least significant difference
SD: Standard deviation
MW: Molecular weight
kDa: Kilodalton
UTR: Untranslated regions
*A. thaliana*: *Arabidopsis thaliana*
IAA: Indole-3-acetic acid
ABA: Abscisic acid
MYB: Myeloblastosis
ARE: Antioxidant Response Element
ABRE: Abscisic Acid-Responsive Element
ERE: Ethylene-Responsive Element
bZIP: Basic leucine zipper
NF-YA: Nuclear Factor Y A subunit complex
DREB2A: Dehydration-Responsive Element Binding protein 2A

## References

[1] Song JM, Guan Z, Hu J, Gua C, Yang Z, Wang S, et al. Eight high-quality genomes reveal pan-genome architecture and ecotype differentiation of *Brassica napus*. Nat Plants. 2020;6:34–45. 10.1038/s41477-019-0577-7. PMC6965005 31932676

[2] Mohamed IAA, Shalby N, El-Badri AM, Awad-Allah EFA, Batool M, Saleem MH, Wang Z, Wen J, Ge X, Xu Z, Wang J, Kuai J, Wang B, Zhou G, Fu T. Multipurpose uses of rapeseed (*Brassica napus* L.) crop (food, feed, industrial, medicinal, and environmental conservation uses) and improvement strategies in China. J Agric Food Res. 2025;20:101794. 10.1016/j.jafr.2025.101794.

[3] Miguel GG, Cellini F, Fotopoulos V, Balestrini R, Arbona V. New approaches to improve crop tolerance to biotic and abiotic stresses. Physiol Plant. 2022;174:13547. 10.1111/ppl.13547. PMC9290814 34480798

[4] Qi H, Xia FN, Xiao S, Li J. TRAF proteins as key regulators of plant development and stress responses. J Integr Plant Biol. 2022;64:431–48. 10.1111/jipb.13182. 34676666

[5] Park HH. Structure of TRAF family: current understanding of receptor recognition. Front Immunol. 2018;9:1999. 10.3389/fimmu.2018.01999. PMC6125299 30214450

[6] Sunnerhagen M, Pursglove S, Fladvad M. The new MATH: homology suggests shared binding surfaces in meprin tetramers and TRAF trimers. FEBS Lett. 2002;530:1–3. 10.1016/S0014-5793(02)03330-6. 12387856

[7] Zapata JM, Martínez-García V, Lefebvre S. Phylogeny of the TRAF/MATH domain. Adv Exp Med Biol. 2007;597:1–24. 10.1007/978-0-387-70630-6_1. 17633013

[8] Pineda G, Ea CK, Chen ZJ. Ubiquitination and TRAF signalling. Adv Exp Med Biol. 2007;597:80–92. 10.1007/978-0-387-70630-6_7. 17633019

[9] Zhao L, Huang Y, Hu Y, He X, Shen W, Liu C, et al. Phylogenetic analysis of *Brassica rapa* MATH-domain proteins. Curr Genomics. 2013;14:214–23. 10.2174/1389202911314030007. PMC3664471 24179444

[10] Kushwaha HR, Joshi R, Pareek A, Singla-Pareek SL. MATH-domain family shows response toward abiotic stress in *Arabidopsis* and rice. Front Plant Sci. 2016;7:923. 10.3389/fpls.2016.00923. PMC4923191 27446153

[11] Dai Y, Ma S, Guo Y, Zhang X, Liu D, Gao Y, et al. Evolution and expression of the meprin and TRAF homology domain-containing gene family in Solanaceae. Int J Mol Sci. 2023;24(10):8782. 10.3390/ijms24108782. PMC10218331 37240124

[12] Kumar S, Stecher G, Tamura K. MEGA7: molecular evolutionary genetics analysis version 7.0 for bigger datasets. Mol Biol Evol. 2016;33:1870–4. 10.1093/molbev/msw054. PMC8210823 27004904

[13] Chen C, Chen H, Zhang Y, Thomas HR, Frank MH, He Y, et al. TBtools: an integrative toolkit developed for interactive analyses of big biological data. Mol Plant. 2020;13:1194–202. 10.1016/j.molp.2020.06.009. 32585190

[14] Krzywinski M, Schein J, Birol I, Connors J, Gascoyne R, Horsman D, et al. Circos: an information aesthetic for comparative genomics. Genome Res. 2009;19:1639–45. 10.1101/gr.092759.109. PMC2752132 19541911

[15] Chao HY, Li T, Luo CY, Huang HL, Ruan YF, Li XD, et al. BrassicaEDB: a gene expression database for *Brassica* crops. Int J Mol Sci. 2020;21(16):5831. 10.3390/ijms21165831. PMC7461608 32823802

[16] Yang ZQ, Wang SB, Wei LL, Huang YM, Liu DX, Jia YP, et al. BnIR: A multi-omics database with various tools for *Brassica napus* research and breeding. Mol Plant. 2023;16:775–89. 10.1016/j.molp.2023.03.007. 36919242

[17] Livak KJ, Schmittgen TD. Analysis of Relative Gene Expression Data Using Real-Time Quantitative PCR and the 2−∆∆CT Method. Methods. 2001;25:402–8. 10.1006/meth.2001.1262. 11846609

[18] Li J, Li Y, Wang R, Fu J, Zhou X, Fang Y, et al. Multiple functions of MiRNAs in *Brassica napus L*. Life. 2022;12:1811. 10.3390/life12111811. PMC9694376 36362967

[19] Marand AP, Eveland AL, Kaufmann K, Springer NM. Cis-regulatory elements in plant development, adaptation, and evolution. Annu Rev Plant Biol. 2023;74:111–37. 10.1146/annurev-arplant-070122-030236. PMC9881396 36608347

[20] Mehmood SS, Lu G, Luo D, Hussain MA, Raza A, Zafar Z, et al. Integrated analysis of transcriptomics and proteomics provides insights into the molecular regulation of cold response in *Brassica napus*. Environ Exp Bot. 2021;187:104480. 10.1016/j.envexpbot.2021.104480.

[21] Chen HX, Wang TP, He XN, Cai X, Lin RM, Liang JL, et al. BRAD V3.0: an upgraded Brassicaceae database. Nucleic Acids Res. 2022;50:D1432–41. 10.1093/nar/gkab1057. PMC8728314 34755871

[22] Zhu WZ, Wu DZ, Jiang LX, Ye LZ. Genome‑wide identification and characterization of SnRK family genes in *Brassica napus*. BMC Plant Biol. 2020;20:287. 10.1186/s12870-020-02484-3. PMC7310057 32571241

[23] Sehrish S, Sumbal W, Xie ML, Zhao CJ, Zuo R, Gao F, et al. Genome‑wide identification and characterization of SET domain family genes in *Brassica napus L.*. Int J Mol Sci. 2022;23:1936. 10.3390/ijms23041936. PMC8879272 35216050

[24] Cosson P, Sofer L, Le QH, Léger V, Schurdi-Levraud V, Whitham SA, et al. RTM3, which controls long-distance movement of potyviruses, is a member of a new plant gene family encoding a meprin and TRAF homology domain containing protein. Plant Physiol. 2010;154:222–32. 10.1104/pp.110.155754. PMC2938151 20584941

[25] Jurani´c M, Srilunchang KO, Krohn NG, Leljak-Levanic D, Sprunck S, Dresselhaus T. Germline-specific MATH-BTB substrate adaptor MAB1 regulates spindle length and nuclei identity in maize. Plant Cell. 2012;24:4974–499. 10.1105/tpc.112.107169. PMC3556970 23250449

[26] Gingerich DJ, Gagne JM, Salter DW, Hellmann H, Estelle M, Ma L, et al. Cullins 3a and 3b assemble with members of the broad complex/tramtrack/bric‐a‐brac (BTB) protein family to form essential ubiquitin‐protein ligases (E3s) in *Arabidopsis*. J Biol Chem. 2005;280:18810–21. 10.1074/jbc.M413247200. 15749712

[27] Lechner E, Leonhardt N, Eisler H, Parmentier Y, Alioua M, Jacquet H, et al. MATH/BTB CRL3 receptors target the homeodomain-leucine zipper ATHB6 to modulate abscisic acid signalling. Dev Cell. 2011;21:1116–28. 10.1016/j.devcel.2011.10.018. 22172674

[28] Zhou H, Zhao J, Cai J, Patil SB. Ubiquitin‐specific proteases function in plant development and stress responses. Plant Mol Biol. 2017;94:565–76. 10.1007/s11103-017-0633-5. 28695315

[29] Tan X, Zhou Y, Cheng Y, Raza A, Lv Y, Chen Y, et al. Identification and functional characterization of the *BnRAP2.3.2* promoter in rapeseed (*Brassica napus L.*) for waterlogging stress tolerance. Plant Stress. 2025;15:100787. 10.1016/j.stress.2025.100787.

[30] Hu H, Jiang Y, Liu C, Zhang Y, Chen M, Liu Z. Genome-Wide Identification and Characterization of Basic Pentacysteine (BPC) Gene Family in *Brassica napus*. Plants. 2025;14(7):1136. 10.3390/plants14071136. PMC11991588 40219204

[31] Zhao G, Cheng Q, Zhao Y, Wu F, Mu B, Gao J, et al. The abscisic acid–responsive element binding factors MAPKKK18 module regulates abscisic acid–induced leaf senescence in *Arabidopsis*. J Biol Chem. 2023;299(4):103060. 10.1016/j.jbc.2023.103060. PMC10166789 36841482

[32] Chen S, Qiu Y, Lin Y, Zou S, Wang H, Zhao H, Shen S, Wang Q, Wang Q, Du H, Li J, Qu C. Genome-wide identification of B-box family genes and their potential roles in seed development under shading conditions in rapeseed (*Brassica napus L*.). Int J Mol Sci. 2024;13(16):2226. 10.3390/plants13162226. PMC11359083 39204662

[33] Cai YM, Yu XM, Hu SN, Yu J. A brief review on the mechanisms of miRNA regulation. Geno Prote & Bio. 2009;7:147–54. 10.1016/S1672-0229(08)60044-3. PMC5054406 20172487

[34] Bai JF, Wang YK, Wang P, Duan WJ, Yuan SH, Sun H, et al. Uncovering male fertility transition-responsive miRNA in a wheat photo-thermosensitive genic male sterile line by deep sequencing and degradome analysis. Front Plant Sci. 2017;8:1370. 10.3389/fpls.2017.01370. PMC5550412 28848574

[35] Taghvaei MM, Lahiji HS, Bakhtiarizadeh MR, Golafazani MM. Bioinformatics analysis of microRNAs related to cold stress and their effects on proteins associated with fatty acids metabolism in rapeseed (Brassica napus L). Crop Biotechnol. 2019;9:41–58. 10.30473/CB.2019.48213.1780.

[36] Ewan R, Pangestuti R, Thornber S, Craig A, Carr C, O’Donnell L, et al. Deubiquitinating enzymes AtUBP12 and AtUBP13 and their tobacco homologue NtUBP12 are negative regulators of plant immunity. New Phytol. 2011;191:92–106. 10.1111/j.1469-8137.2011.03672.x. 21388379

[37] Cui X, Lu F, Li Y, Xue Y, Kang Y, Zhang S, et al. Ubiquitin-specific proteases UBP12 and UBP13 act in circadian clock and photoperiodic flowering regulation in Arabidopsis. Plant Physiol. 2013;162:897–906. 10.1104/pp.112.213009. PMC3668078 23645632

[38] Morimoto K, Ohama N, Kidokoro S, Mizoi J, Takahashi F, Todaka D, et al. BPM-CUL3 E3 ligase modulates thermotolerance by facilitating negative regulatory domain-mediated degradation of DREB2A in *Arabidopsis*. Proc Natl Acad Sci U S A. 2017;114:E8528–36. 10.1073/pnas.1704189114. PMC5635875 28923951

[39] Skiljaica A, Lechner E, Jagić M, Majsec K, Malenica N, Genschik P, et al. The protein turnover of Arabidopsis BPM1 is involved in regulation of flowering time and abiotic stress response. Plant Mol Biol. 2020;102:359–72. 10.1007/s11103-019-00947-2. 31848919

[40] Huang S, Chen X, Zhong X, Li M, Ao K, Huang J, et al. Plant TRAF proteins regulate NLR immune receptor turnover. Cell Host Microbe. 2016;19:204–15. 10.1016/j.chom.2016.01.005. 26867179

[41] Qi H, Xia FN, Xie LJ, Yu LJ, Chen QF, Zhuang XH, et al. TRAF family proteins regulate autophagy dynamics by modulating AUTOPHAGY PROTEIN6 stability in *Arabidopsis*. Plant Cell. 2017;29:890–911. 10.1105/tpc.17.00056. PMC5435438 28351989

[42] Linden KJ, Callis J. The ubiquitin system affects agronomic plant traits. J Biol Chem. 2020;295:13940–55. 10.1074/jbc.REV120.011303. PMC7535913 32796036

[43] Leljak‐Levanić D, Juranić M, Sprunck S. De novo zygotic transcription in wheat (*Triticum aestivum* L.) includes genes encoding small putative secreted peptides and a protein involved in proteasomal degradation. Plant Reprod. 2013;26:267–85. 10.1007/s00497-013-0229-4. 23912470

